# Electrochemical Redox Cycling Behavior of Gold Nanoring Electrodes Microfabricated on a Silicon Micropillar

**DOI:** 10.3390/mi14040726

**Published:** 2023-03-24

**Authors:** Haocheng Yin, Chao Tan, Shabnam Siddiqui, Prabhu U. Arumugam

**Affiliations:** 1 School of Microelectronics, Xidian University, Key Laboratory of Wide Band-Gap Semiconductor Materials and Devices of China, Xi’an 710071, China; 2 Institute for Micromanufacturing, Louisiana Tech University, Ruston, LA 71272, USA; 3 Department of Chemistry and Physics, Louisiana State University Shreveport, Shreveport, LA 71101, USA

**Keywords:** Nanoelectrodes, concentric, micropillar, electrochemistry, redox cycling, nanofabrication

## Abstract

We report the microfabrication and characterization of concentric gold nanoring electrodes (Au NREs), which were fabricated by patterning two gold nanoelectrodes on the same silicon (Si) micropillar tip. Au NREs of 165 ± 10 nm in width were micropatterned on a 6.5 ± 0.2 µm diameter 80 ± 0.5 µm height Si micropillar with an intervening ~ 100 nm thick hafnium oxide insulating layer between the two nanoelectrodes. Excellent cylindricality of the micropillar with vertical sidewalls as well as a completely intact layer of a concentric Au NRE including the entire micropillar perimeter has been achieved as observed via scanning electron microscopy and energy dispersive spectroscopy data. The electrochemical behavior of the Au NREs was characterized by steady-state cyclic voltammetry and electrochemical impedance spectroscopy. The applicability of Au NREs to electrochemical sensing was demonstrated by redox cycling with the ferro/ferricyanide redox couple. The redox cycling amplified the currents by 1.63-fold with a collection efficiency of > 90% on a single collection cycle. The proposed micro-nanofabrication approach with further optimization studies shows great promise for the creation and expansion of concentric 3D NRE arrays with controllable width and nanometer spacing for electroanalytical research and applications such as single-cell analysis and advanced biological and neurochemical sensing.

## 1. Introduction

Nanoelectrodes (NEs) with their unique size-dependent properties are widely utilized as detectors in several areas of (bio-) electrochemistry, which includes nanoelectrode lithography, single cell analysis, scanning electrochemical microscopy (SECM), and many others [1,2,3,4,5]. NEs are superior to microelectrodes in terms of their higher signal-to-noise ratio (SNR), higher sensitivity, lower detection limits, smaller volume probed, higher mass transport rates owing to radial-type diffusion, more rapid detection, higher spatial-temporal resolution, and the ability to apply them to highly resistive media [6,7,8,9].

Using advanced micro- and nano-fabrication techniques, NEs of various geometries (e.g., disk, band, hemispherical, interdigitated) have been widely fabricated and applied in fundamental electrochemical and bio-electrochemical sensing applications [10,11,12,13]. However, the NEs’ nanoscale geometry makes the detection sensitivity quite high. They also generate too low currents that are difficult to observe and detect. To solve this problem, ring-shaped and band NEs, which have widths in the nanometer range and lengths in the micrometer range, have been developed. The novel nanoring electrodes have the advantages of both nanoelectrodes and microelectrodes and are widely applied in the electrochemistry and bio-electrochemistry fields [14,15,16]. In previous studies, NEs have been commonly applied by increasing faradaic currents (i.e., detection signals) to fully employ the benefits of NE’s electrochemical behavior. Even though a single NE can be quite beneficial owing to its nanoscale dimensions [17,18,19], multiple NEs with only a few hundred nanometers spacing in an interdigitated electrode array (IDA) format and with overlapping redox concentration profiles were found to be attractive in many electroanalytical applications [20,21]. In such nano IDA systems, one of the forms of redox species is generated at one NE (called a “generator”), which is then collected at another nearby NE (called a “collector”), where it is converted into the other form of the redox species. This electrochemical cycling is called the redox cycling (RC) effect. The RC effect is effectively used in sensor applications for signal amplification and enhanced detection selectivity in the presence of interferants [6].

In a redox reaction, electrons are transferred between two compounds. Molecule A loses electrons and oxidizes to form molecule B, while molecule B reduces back to A by gaining electrons. The electron in a redox reaction is donated by electron donors or transferred to electron acceptors. The compounds are called reductants when donating an electron or being oxidized and oxidants when they are reduced or receive an electron from other compounds [22,23]. The pair of compounds that are reduced and oxidized is known as a “redox couple” [24,25]. Redox cycling is a series of repetitively coupled reduction and oxidation reactions, which can measure the affinity of a molecule for electrons. As one of the most investigated electrochemical phenomena, the RC effect has been observed through various closely spaced NE geometries, such as concentric-band electrodes, thin-layer cells, and ring-disk electrodes [20,22,23,24,25,26].

The geometries of traditional NEs (eg concentric band, ring-disk) for redox cycling measurements that have been well-developed for fundamental electrochemical sensing, are typically microfabricated as a planar array and in a cavity format. Planar array NEs are microfabricated through multiple metal patterning procedures such as photolithography, physical and chemical vapor deposition, and dry/wet chemical etching processes. Cavity NEs are typically created by applying multiple microfabrication etching processes in sequence on a sandwich architecture. Outer and inner disk-ring-shaped electrodes are generated during the etching steps. The advantages of such processes include: (1) the width of the ring-disk NEs can be easily controlled through the etching mask; (2) the gap thickness between the two NEs can be precisely controlled via the thickness of the insulator deposition steps using various techniques (e. g., plasma-enhanced chemical vapor deposition, atomic layer deposition). Despite their broad potential applicability, their geometries on either planar and/or cavities have posed severe limitations in terms of signal saturation, which are caused by (1) a smaller radial diffusion area, (2) analytes captured in the cavity, (3) poor cell viability and/or proliferation caused by 2D geometries, and (4) increased resistance for the analytes to reach the recessed NEs in a microfluidic system that dominates the field of micro electroanalytical applications [27,28,29,30,31]. One of the solutions is to pattern the band NEs on a three-dimensional (3D) microstructure (i.e., a 3D NRE). Such 3D micro- and nano-structures have demonstrated excellent cell attachment and growth ability and superior mass transport rates and electrochemical behavior [32,33,34,35]. However, no NE fabrication technique has yet been demonstrated that properly integrates 3D NEs for a redox cycling study.

**Figure 1:**
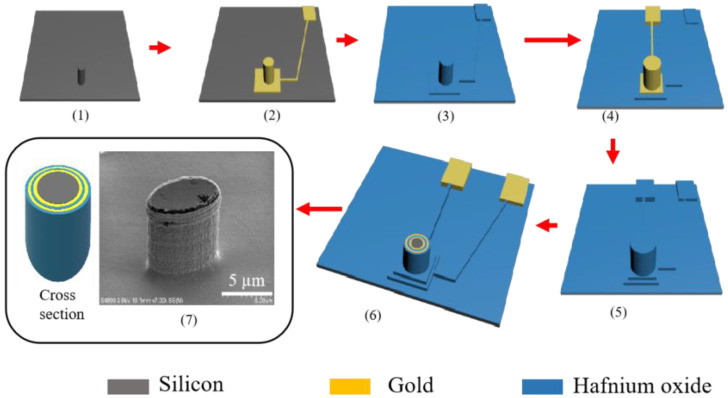
Au NREs fabrication flow diagram. (**1**) ICP dry etching of silicon micropillar; (**2**) inner Au electrode patterning (generator electrode); (**3**) ALD deposition of HfO_2_ insulation layer; (**4**) outer Au electrode patterning (collector electrode); (**5**) ALD deposition of outer HfO_2_ insulation layer; (**6**) defining the Au NRE via etching HfO_2_ − Au − HfO_2_ − Au sequentially; (**7**) SEM image of the NREs on the micropillar.

In a previous study, our group reported the microfabrication and characterization of a single gold (Au) NRE patterned on a silicon micropillar, providing the 3D electrode geometry with a superior signal-to-noise ratio (SNR) of up to 2500 that is 10-fold greater than that of the traditional band NEs fabricated by etching through the surface of a sandwich structure [36]. We demonstrated the applicability of Au NREs to electrochemical sensing of lead, a neurotoxin at 100 ppb levels. Also, by surface modification with multiwalled carbon nanotubes, dopamine, a neurochemical implicated in various brain disorders, was detected at a sensitivity as low as 100 nM with 1000-fold selectivity versus common interferents. The single NRE work laid a solid foundation to design, fabricate, and test a double NRE system for redox cycling applications, the central goal of this work. Here, we report the first study of 165 nm wide Au nanoring nano band electrodes (Au NREs) patterned on the tip of a 7.2 µm diameter (*φ*) × 8.0 µm high Si micropillar and separated by a 100 nm thick insulation layer (Figure 1). In this work, we focused on the microfabrication of two concentric NREs on a single silicon micropillar structure. The proposed fabrication method can be easily extended to more than two NREs and with other thin film electrode materials to achieve a highly multiplexed nano-sensor platform. The fabricated NREs were characterized by scanning electron microscopy (SEM), energy dispersive spectroscopy (EDS), cyclic voltammetry (CV), surface profilometry, confocal microscopy, and electrochemical impedance spectroscopy (EIS).

## 2. Materials and Methods

### 2.1. Microfabrication Processing

Silicon micropillars microfabricated on 1.6 cm square chips were obtained by dicing 4-inch undoped silicon wafers (University Wafer Inc, Boston, MA 02127, USA). The chips were cleaned in Buffered Oxide Etchant (BOE) (10:1) for 1 min, rinsed with DI water for 1 min, and baked at 210°C for 2 min. After the chips had cooled down, they were spin-coated with photoresist, PR (PR 1813, Microchem, Inc, Westborough, MA 01581, USA) at 3000 rpm for 50 s followed by a pre-bake (115 °C, 150 s) and a post-bake (115 °C, 60 s). Then chrome mask 1 was applied for UV exposure with a SUS MicroTech EV 420 Mask Aligner (400 nm, 30 s exposure, WEC contact mode, soft exposure type) to obtain 4 µm diameter circular patterns on the chips. The chips were then immersed in MF-319 developer for 60 s to transfer the patterns from the mask to the photoresist layer. Next, the chips were rinsed in DI water for 30 s and again baked at 115 °C for 60 s. The PR pattern served as a dry-etching hard mask during inductively coupled plasma (ICP) deep reactive ion etching (DRIE), a dry-etching process for the high-aspect-ratio micropillar fabrication (STS HRM ICP) system, with 22 etching cycles, each cycle includes 130 sccm/8.0 s for sulfur hexafluoride (SF_6_), 100 sccm/5.5 s for octafluorocyclobutane (C_4_F_8_), 13 sccm/8.0 s for oxygen (O_2_) that flowed with sulfur hexafluoride, and 600 W, at room temperature) [37,38,39]. After removing the PR, a 150 nm thick Au film with a 25 nm thick chromium adhesion layer was deposited onto the silicon micropillar by an E-beam evaporation technique. Chrome mask 2 was then applied to pattern the Au thin film on the micropillar by adopting a first lift-off process step. To avoid a very thick insulation film and process complexity, hafnium oxide (HfO_2_) was used as the insulating layer between the two Au NREs, due to its excellent dielectric properties [40,41,42]. After the first Au layer was patterned on the pillars, a 100 nm HfO_2_ layer was coated on the micropillar with a Cambridge Fiji Plasma ALD system (deposition rate, 0.9A/cycle; temperature, 200 °C). Chrome mask 3 was used during the second Au layer patterning procedure, which was similar to the previous metal lift-off procedures. After the outer 100 nm HfO_2_ insulator layer was coated, a photoresist wet etching mask was patterned on the chips’ surface with a lithography process and chrome mask 4. The HfO_2_ layers and the Au layers, which were deposited on the tip of the micropillar, were subsequently removed from outside to inside. The HfO_2_ was etched by a 10:1 BOE with a rate of 212 A/s at room temperature (confirmed by confocal microscopy and EDS analysis). The Au exposed on top of the micropillar at this stage behaves as a disk microelectrode. The next step was to etch the gold microdisk and the underlying Cr seed layer very carefully into the final Au NREs. At an etch rate of 364 A/s at room temperature, the Au was etched in gold etchant initially for 41 s (Sigma Aldrich, St. Louis, MO 63178, USA), followed by rinsing in DI water and drying at 80 °C for 60 s. The final NREs were formed by repeating the etching process and removing the inner HfO_2_ film and the inner Au layer. Chromium etchant (Sigma Aldrich, St. Louis, MO 63178, USA) with an etch rate of 40 A/s was then applied to etch the chromium adhesion layer. The devices under fabrication were etched for another 10 s to 30 s until they exhibited appropriate NE behavior as evidenced by microelectrode behavior in a CV voltammogram. Figure 1 illustrates the Au NREs fabrication flow diagram.

### 2.2. Morphology

Micropillar cylindrical geometries were investigated with a field-emission scanning electron microscope (FESEM: Hitachi S-4800, Tokyo, Japan). The film thickness was characterized by surface profilometry (DEKTAK, 150 Profiler, Karlsruhe, Germany). The EDS results were also obtained with the FESEM.

### 2.3. Electrochemical Characterization

Cyclic Voltammetry was carried out with an Autolab potentiostat (PGSTAT 302N, Metrohm Herisau, Switzerland) in a three-electrode configuration including the Au NREs as working electrodes, a saturated calomel reference electrode (SCE, Accumet, Singapore) and a Pt coil (Alfa Aesar, Haverhill, MA 01876, United States) counter electrode. The freshly prepared electrochemical solution that was applied for all the experiments was purged in nitrogen for 10 mins before use. The potentiostat was operated with a Frequency Response Analyzer 2, low current module, and Nova 1.10.3 software. The recorded scanning potential window is between −0.2 V and +0.6 V with a range of scan rates (10 mV/s to 1000 mV/s) for the cyclic voltammograms. The recording window range for the EIS spectra was from 100 kHz and 100 mHz with a 10 mV AC signal amplitude (rms value) at open circuit potential (OCP). The impedance data were analyzed by applying a nonlinear least-squares fit to the appropriate theoretical model represented by an equivalent electrical circuit set. The CV and EIS analysis was performed in 5 mM K_4_Fe(CN)_6_/ 5 mM K_3_Fe(CN)_6_ in 1 M KCl. The redox cycling effect was measured in 5 mM K_3_Fe(CN)_6_ in 1 M KCl and recorded between −0.2 V and +0.6 V, where the inner NRE (i.e., NRE 1) was swept at multiple scan rate (10 mV/s to 500 mV/s) and the outer NRE (i.e., NRE 2) was held +0.5 V. The non-redox cycling effect was measured in 5 mM K_3_Fe(CN)_6_ in 1 M KCl and recorded between −0.2 V and +0.6 V, where NRE 1 was swept at multiple scan rates (10 mV/s to 500 mV/s) and NRE 2 was floating. Out of a total of 20 chips (each chip containing three NREs), 40 NREs were tested at random. Eleven NREs were found to be unsuitable for further characterization due to poor or no electrical connections. The other 29 NREs were used for the various studies reported herein.

## 3. Results and Discussion

### 3.1. Surface Characterization of Si Micropillar and Au NREs

**Figure 2:**
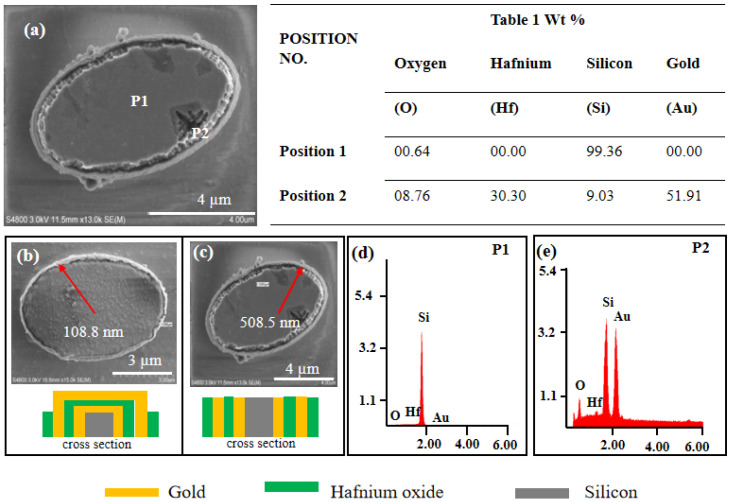
SEM images showing a top view of the Au NREs, (**a**) after all the wet-etching processes are complete, (**b**) width measurements of the outer layer thickness after removing the first HfO_2_ layer. (**c**) width measurement of the outer layer thickness after the final fabrication process. (**d**,**e**) EDS plots quantifying the elemental composition of the silicon micropillar’s tip center (Position 1), tip edge that includes the two Au layers and two HfO_2_ layers (Position 2). The table in the figure lists the elemental wt%’s in P1 and P2 separately.

From the SEM images (Figure 2a), the average radius (r) of the fabricated chip containing two NREs was determined to be 3.5 µm with an interelectrode spacing of 100 nm, which is based on the thickness of the ALD HfO_2_ deposition process. The average height of the electrode is 8 µm (d ≈ 2.25r). From the SEM images, it is hard to distinguish the two adjacent Au NREs located between the HfO_2_ layers and micropillar. Due to the edge effect for SEM observations, the ultrathin HfO_2_ layer is also undetectable. To verify the exposure of the NREs after the gold etching process, an EDS technique was applied at different locations close to the micropillar edge to confirm the presence of the Au NREs along the perimeter of the micropillar (Figure 2d,e). Position 1 located on the tip of the pillar structure has a silicon element weight percentage as high as 99.63 wt%, which proves that all the layers on the pillar’s tip have been completely etched. Position 2 located at the edge of the tip has 8.76% oxygen, 9.03% silicon, 30.30% hafnium, and 51.91% Au (all in wt%). The EDS results proved that: First, the cover layers on the tip of the pillar had been completely removed. Second, the Au NREs have been exposed on the edge of the pillar’s tip. Third, the sidewall has been well protected during the etching process.

Considering the thickness of all the covered layers on the micropillar, the Au film thickness is 150 nm ± 10 nm (n = 3) based on the control sample that was partially wet etched and then measured with a surface profilometer (DEKTAK, 150 Profiler, Karlsruhe, Germany). After the outer HfO_2_ layer that covers the tip is etched, the thickness of the outer HfO_2_ layer located on the pillar’s sidewall was found to be 108.8 ± 5 nm via SEM (Figure 2b). The four outer layers’ total thickness for the fabricated NREs was 508.5 ± 10 nm (Figure 2c). The measurement value is close to the layers’ total theoretical thickness as anticipated. The SEM and EDS results demonstrate the fabrication of high-quality concentric Au NREs.

### 3.2. CV Theory and Analysis

The EDS results demonstrate that the NREs have been fabricated with high quality during wet etching and are ready for electrochemical studies. A Cyclic Voltammetry (CV) technique was applied to investigate the general electrochemical behavior of the NREs such as their steady-state radial diffusion dominant faradaic current responses (*I*_ss_, A). The *I*_ss_ can be described by Equation (1) [43]:

(1) $$I_{ss}=\frac{nFC_{b}}{R_{MT}}$$

where *C*_b_ is the bulk concentration of redox species in the solution with units of mM and *R*_MT_ is the mass-transfer resistance with units of m/s. *F* and *n* are Faraday’s constant (96,485 °C mol^−1^) and the number of transferred electrons respectively. For an NRE, *R*_MT_ and a time component *t* (s) can be introduced by Equations (2) and (3) [44]:

(2) $$R_{MT}=\frac{In\frac{64Dt}{w^{2}}}{2\pi Dl}$$

(3) $$t=\frac{RT}{Fv}$$

where *D* is the diffusion coefficient of redox species (cm^2^ s^−1^), and *w* represents the width of the NRE (nm). *l* and *T* represent the length of the NRE (µm) and the temperature (K) separately. The theoretical *I*_ss_ for the NRE is calculated by Equation (4), which is formed by applying Equation (3) and Equation (2) to Equation (1). Equation (4) has application in mathematical analysis and will be discussed later.

(4) $$I_{ss}=\frac{2\pi nFC_{b}Dl}{In\frac{64Dt}{w^{2}}}$$

**Figure 3:**
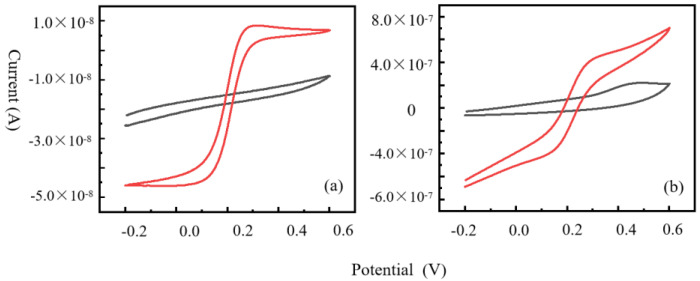
Cyclic voltammograms of inner (**a**) NRE 1 and (**b**) outer NRE 2 at a 100 mV/s scan rate. Signal (red curve) is generated with a 5 mM K_4_Fe(CN)_6_/ 5 mM K_3_Fe(CN)_6_ redox couple in 1M KCl. The background current (black curve) is generated in 1 M KCl.

The CV results of the inner NRE 1 and the outer NRE 2 in the electrolyte (i.e., background or charging current) and in a 5 mM redox couple (i.e., faradaic current) are displayed in Figure 3a and Figure 3b, respectively. For NRE 1, the background and faradaic currents were 0.5 nA and 5.8 nA (Figure 3a), respectively, with an SNR of 12. For NRE 2, the background and faradaic currents were 100 nA and 500 nA (Figure 3a) with an SNR of 5.

The data suggest that NRE 2 is somewhat larger than NRE 1 even though their approximate size and geometry are similar, i.e., only a few 100 nm different.

**Figure 4:**
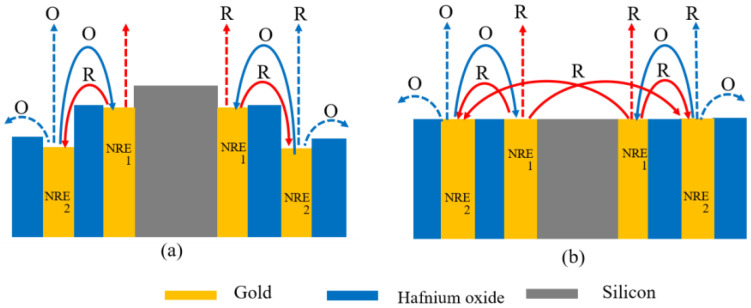
Schematic of analyte diffusion paths. Cross-sectional view of the (**a**) actual geometry of Au NREs, (**b**) designed/expected/theoretical geometry of Au NREs. Legends: Au (yellow), silicon (grey), HfO_2_ (blue). “R” represents reduction production and “O” represents oxidation production. Analytes lost due to random diffusion (dashed curve); analytes partaking in RC (solid curve); reduction product (red curve); oxidation product (blue curve).

To understand the large signal variation between the NREs, we calculated the expected steady-state current from a single NRE by applying Equation (4). The calculations based on the SEM data (*d*_NRE 1_=7.02 μm, *d*_NRE 2_=7.43 μm) indicate that the *I*_SS_ (inner) value should be about 6.3 nA to 7.6 nA and that the 5.8 nA measured current signal obtained was in the reasonable range of *I*_ss_. The small difference between the current values could be due to processing variations, which have been detailed in our previously published work [36]. In this work, we observed that the last stage of the multi-step fabrication process includes several wet etching steps. The wet etching effect would be enhanced several times at the nanoscale dimensions of these devices. Ideally, an inlaid NRE geometry with no recess or trench is desirable during microfabrication (Figure 4b). The CV data suggests that the *I*_ss_ values and the shape of the cyclic voltammograms vary among the NREs and this could be due to the uncontrollable variations during the final gold wet etching process. Under-etching of gold will result in a disk microelectrode geometry instead of an NRE, and over-etching of gold will result in an NRE located in a trench (Figure 4a). Since the gold is slightly over-etched to ensure the complete development of the NRE, the formation of a nanotrench with some depth (*d*) is expected. The most desirable outcome will be a well-defined NRE with the lowest trench depth. Importantly, the trench caused by over-etching could increase the solution and electrical resistance, which leads to a reduction in current. However, the voltammogram of the inner NRE 1 (5.8 nA) demonstrated nearly ideal nanoelectrode behavior, i.e., no leakage currents. Conversely, the experimentally measured current of the outer NRE 2 (500 nA) is much greater than the theoretical value. This proves that the nanoelectrode sidewall was not well protected and has pores (or porosity) exposing the NE to the bulk solution. During the final fabrication procedures, the PR mask is removed with acetone and with a subsequent DI water rinse, which forms surface defects such as pores on the electrode sidewall’s insulation layer. These defects increased the total exposed area of the outer NRE 2, which led to the observed leakage currents. When the outer NRE 2 is the generator, the reduction product diffuses outward into the bulk solution instead of diffusing toward the inner collector NRE 1 (Figure 4). The CV results demonstrate that the outer NRE 2 is not well protected, which means more analytes will be generated from the electrode defect on the sidewall and they will diffuse back into solution instead of collecting at the inner NRE 1. Due to the loss of the reduction product, the collection efficiency will be much lower than the expected value when NRE 2 is the generator. To address this shortcoming and improve the collection efficiency, NRE 1 was chosen as the generator.

### 3.3. EIS Analysis

**Figure 5:**
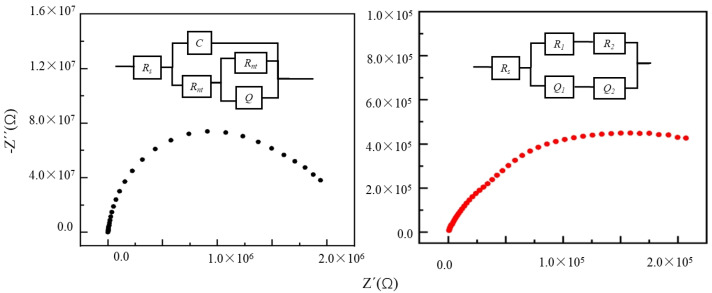
Nyquist plot of inner Au NRE 1 (black) and outer Au NRE 2 (red). The equivalent circuits are shown in the Inset. The electrolyte is 5 mM Fe (CN)_6_^3-/4-^ in 1M KCl, tested at 10 mV amplitude, OCP, across a frequency range of 0.1 Hz-100 KHz.

To further investigate the effect of microfabrication on the electrochemical behavior of the NREs, we employed electrochemical impedance spectroscopy (EIS) [45,46,47,48,49,50,51]. As mentioned in our previous published work [36], NREs show a semicircular arc in representative Nyquist plots (real vs. imaginary parts of the impedance) with no scattered data points, i.e., no noise, which is a unique electrochemical property of NEs (Figure 5). 

A similar demonstration on nanocrystalline diamond nanoelectrode arrays has also been reported, which also shows a similar impedance spectrum [46]. The charge transfer resistance (*R*_ct_) of the kinetically controlled NRE is shown in Equation (6), which is represented by the diameter of the semicircle plot and is inversely proportional to the exchange current (*i*_0_) that is introduced by Equation (5) [52]. The semicircle arc-type behavior of the impedance suggests that *i*_0_ is controlled by the electrode kinetic, particularly at low and very low frequencies.

*i*_0_ = *nFkAC*_b_ (5)

(6) $$R_{ct}=\frac{R_{G}T}{nFi_{0}}$$

where *A* is the NRE area, *F* is Faraday’s constant (9.64853 × 10^4^ C/mol), *k* is the standard rate constant (cm s^−1^), *T* is the temperature (298K), and *R*_G_ is the gas constant (8.314 J/mol. K). From the EIS results, the fitted circuit for NRE 1 is [*R*_s_(*C*[*R*_ct_(*R*_nt_*Q*)])] as was previously reported in [36], which includes the following circuit elements: solution resistance (*R*_s_), a double-layer capacitance (*C*), *R*_ct_, a constant phase element (CPE, *Q*), and the solution resistance due to the heterogenous region (*R*_nt_). The estimated % error of the circuit elements is less than 12%. This circuit is like those used to detect defects or pores due to the delamination of surface coatings. These components of the fitted circuit, which includes CPE, *R*_nt_, *C*, and *R*_ct_, suggest that the NRE surface comprises a mixture of homogenous and heterogenous regions, which represents the complex structure of the nanotrench formed during the wet etching stage of the fabrication. The nanotrench region is represented by CPE and *R*_nt_ and the homogenous region is represented by *C* and *R*_ct_. The fitted circuit model for NRE 2 is [*R*_s_(RQ)(RQ)], which is a combination of two circuits contributing to an overall impedance and each corresponds to the two different regions that have varying electrochemical activity. The two regions are the top nanoband NRE 2 and the surrounding nanotrench formed between the HfO_2_ layers (Region 1) and the NRE 2 that are exposed to the outside bulk solution due to the poor insulating HfO_2_ layer (Region 2). The total current and the corresponding electrochemical activity vary in each region due to differences in the geometrical structure of the trenches and pores. The circuit elements are the solution resistance (*R*_s_), two constant phase elements (*Q*_1_, *Q*_2_), and two solution resistances. We used EIS as a useful investigative tool to optimize the wet etching process and to achieve high analyte sensitivity and excellent SNR because the actual values of the circuit elements provide critical insights into the formation of the Au NREs during microfabrication [53,54]. In our previous work [36], we applied the EIS technique and found that the NREs that exhibit a large *R*_ct_ contribution from the nanotrench region also exhibit a lower *i*_0_ due to a reduction in their effective electrode area. As expected, the *R*_ct_ of each of the NREs is different. This difference is mainly due to the inherent variations in the NRE geometry as anticipated during the multi-step microfabrication process. The NREs that exhibit a large *R*_ct_ contribution from the nanotrench region also exhibit a lower *i*_0_ due to a reduction in their effective electrode area. As the *i*_0_ decreases, *R*_ct_ increases. For example, for one of the NRE 1 NREs with *R*_ct_ = 14.5 KΩ, *R*_nt_ = 18.9 MΩ and *Q* = 8 nMho (*N* = 0.871), the experimental *i*_0_ obtained from Equation (6) is 1.2 nA. However, the theoretical *i*_0_ value from Equation (5) is 1.84 nA (*k* = 0.1 cm/s [55], *A* = 3.83E08 cm^2^, *C*_b_ = 5 mM), greater than the experimental value. This suggests that the whole of the actual electrode area in the microfabricated NRE is not electrochemically active. This again suggests that the NRE is in the trench. Whereas for the NREs with smaller effective areas, they should be in trenches with large “d” values. The EIS results of NRE 2 show that the *R*_1_*Q*_1_ and *R*_2_*Q*_2_ are 24.9 KΩ, 363 nMho (*N* = 0.664) and 213 KΩ, 1470 nMho (*N* = 0.523), respectively, for the two regions, suggesting that more electrode area is exposed to the solution and the charging current will be high, which is confirmed by the cyclic voltammograms. The large charging current of NRE 2 is due to the over-exposed Au layer. After the etching process is completed, the protective PR mask is removed by rinsing the fabricated chip with acetone. The extremely thin insulation layer and the weak adhesion between the Au and HfO_2_ layers led to the surface layer cracking during the rinse procedure. The EIS data indicates that the two regions for the outer NRE 2 also conform with the experimental results. This demonstrates that by controlling the microfabrication parameters and, thus, their geometry, NREs with improved electrochemical behavior can be realized. However, in the actual processing, the NRE tip patterning is based on a wet etching process, which is inherently hard to control. Based on the EIS results, we considered a dry etching process as an alternative. However, dry etching HfO_2_, which is very resistant to corrosion, will lead to a much more complex electrode structure causing further damage to the electrode tip, especially on 3D pillar geometries. Thus, wet etching is more suitable to fabricate concentric NREs as reported in this work despite the geometry variations implied by the more isotropic wet etch process.

### 3.4. Redox Cycling

When considering the design of RC experiments, with the outer NRE 2 as the collector electrode, the over-exposed electrode regions will not affect the inner NRE 1 generator electrode, since the two NREs are very closely spaced, and therefore the measurement of current is affected by shorter diffusion paths and times. Conversely, if the outer NRE 2 is the generator electrode, the over-exposed active regions on the sidewalls could lead to more loss of the reduced product. Thus, we have chosen the outer NRE 2 as the collector, which would be floating in non-RC mode and maintained at +0.5 V in RC mode (Figure 4). Thus, we have chosen the inner NRE 1 that only provides half of the possible diffusion directions as the generator. In this way, the whole redox cycling sensor collection efficiency is improved. The inner NRE 1 was set as the generator electrode and the potential was swept between −0.2 V to +0.6 V with multiple scan rates of (10 mV/s to 500 mV/s) in both non-RC and RC modes (Figure 4).

In non-RC mode, we expect a radial diffusion dominant redox reaction at the inner NRE 1 (confirmed by the CV) in the standard ferro/ferricyanide couple. In RC mode, the ferricyanide [Fe (CN)_6_]^3−^ is reduced at the starting negative potentials on NRE 1, as shown in Reaction (1).

[Fe (CN)_6_]^3−∘^ + e^−∘^ → [Fe (CN)_6_]^4−^ (R1)

During the CV measurements, an electric field is established between NRE 1 and NRE 2. The reduced “ferrocyanide” [Fe (CN)_6_]^4−^ is then diffused to NRE 2 and into the bulk solution. At the same time, NRE 2, which is set at an oxidation potential of + 0.5 V, oxidizes the ferrocyanide back (i.e., cycled) to oxidized “ferricyanide”, as shown in Reaction (2).

[Fe (CN)_6_]^4−∞^ → [Fe (CN)_6_]^4−^ + e^−^ (R2)

**Figure 6:**
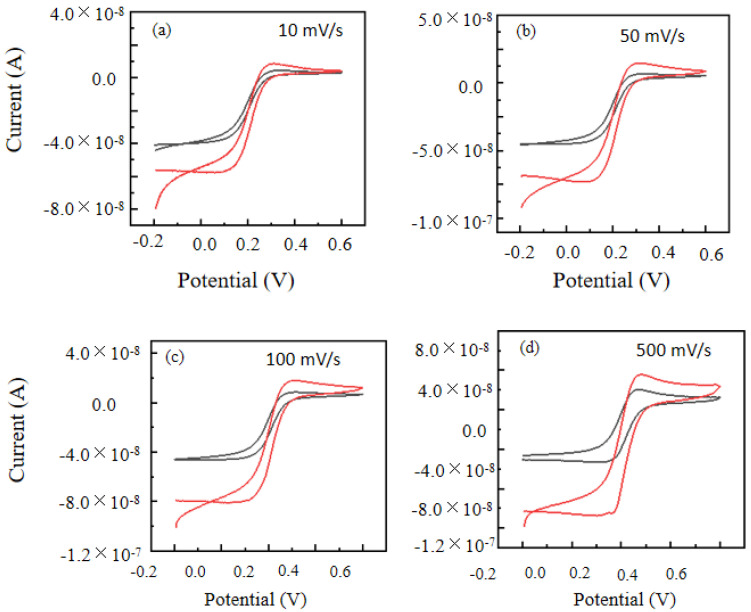
Redox cycling characterization of inner NRE 1 with scan rates (**a**) 10 mV/s, (**b**) 50 mV/s, (**c**) 100 mV/s, (**d**) 500 mV/s. The electrolyte is 5 mM Fe (CN)_6_^3-^ in 1M KCl. The voltammogram of NRE 1 when NRE 2 is floating (black). The voltammogram of NRE 1 when NRE 2 is held at + 0.5 V (red).

The redox-cycled ferricyanide diffuses back to the inner NRE 1 generator electrode to reduce again and amplify the reduction current during the process. Due to the continuously changing (sweeping) potential of NRE 1 between negative (cathodic) and positive (anode), the electric field becomes less effective during certain periods, particularly when the potential at NRE 1 is more positive. When compared to the non-RC mode, the presence of the collector electrode provides more electron transfer events and catalyzes the redox reaction. The RC of the redox couple between the generator and the collector results in current (or signal) amplification. The amplification is calculated from Equation (7):

(7) $$Amplification=\frac{i_{RC}}{i_{non-Rc}}$$

where *i*_RC_ and *i*_non-RC_ are the steady state currents of the generator NRE 1 in RC and non-RC modes, respectively. The experimental results (Figure 6, black) show that the *i*_non-RC_ ranges from 29.2 nA to 65.9 nA when the scan rate is changed from 10 mV/s to 500 mV/s. In RC mode (Figure 6, red), the recorded *i*_RC_ ranges from 47.5 nA to 85.6 nA. The current amplification at NRE 1 calculated from equation (7) is from 1.63 to 1.30-fold (Table 1). The current amplification can be further increased by optimizing the final stages of the wet etch steps so that the trench depths, the loss of the redox species, and the diffusion pathway between the NREs are all kept at a minimum.

The results demonstrate that, as expected, the current amplification is inversely proportional to the scan rate. At a lower scan rate, the redox cycling time will last longer, which leads to a longer diffusion time and redox species will be reduced to a greater degree. Since NRE 2 is set as the collector at a positive potential, the reduced ferrocyanide will diffuse to the collector and oxidize back to ferricyanide, which then diffuses back to the generator and further increases the current amplification signal. Thus, more redox species are contributing to the redox cycling reaction. By applying Equation 8, the collection efficiency of NRE 2 is calculated. The RC effect depends strongly on the collection efficiency, φ, with which the NRE 2 collects the current of species generated at NRE 1. The collection efficiency of the NRE 2, *φ*_2_ is:

(8) $$\varphi_{2}=\frac{i_{2,a}}{i_{1,c}}$$

where *i*_2,a_ and *i*_1,c_ are the anodic and cathodic limiting currents at NRE 2 and NRE 1. It is obvious that the lower scan rate at NRE 1 provides an improved NRE 2 collection efficiency Table 2. When the scan rate of NRE 1 is set to 10 mV/s, a maximum collection efficiency of 90.7% is recorded. The lowest scan rate also provides the maximum amplification, which is 1.63. The low amplification could be due to over-etched NREs as shown in Figure 4a. The length of the redox species diffusion path between the NREs is much longer, causing more loss of the species. As shown in Figure 4a, part of the reduction can be diffused from NRE 1 to NRE 2. After the reduction product is oxidized, not all these oxidized species can diffuse back to NRE 1. Due to the randomness of the diffusion process, only part of the reduction product is collected at the electrode. Since the NREs are designed for bio-electroanalytical sensing applications, the NREs are expected to be surrounded by tissues or cells, which will also limit the reaction volume, and which should further improve the RC effect for the whole system.

## 4. Conclusions

In summary, for the first time, two concentric gold NREs were microfabricated onto a single silicon micropillar and fully characterized. We designed and developed new microprocessing steps to obtain a silicon micropillar with integrated NREs for advanced sensing applications. Several microfabrication steps including the critical wet etching process were optimized to achieve high-quality micropillars and NREs as confirmed by SEM and EDS. The NREs exhibited a high SNR with a steady-state limiting current that is unique to nanoscale electrodes. EIS characterization demonstrated that the NREs were surrounded by nano trenches of varying depths and with some porosity on the outer sidewall of the passivation layer, which is due to the non-optimal wet etching process. This research demonstrates three key benefits: First, the development of a micro-nanofabrication technique that truly integrates band NREs with 3D micropillars and delivers efficient RC detection and current amplification; Second, the demonstration of multiple micropatterned NREs onto a single micropillar with only 100 nm separation via ALD oxide coatings; Third, the demonstration of the new capabilities of concentric 3D NREs in electrochemical sensing applications via redox cycling.

## Figures and Tables

**Table 1:** Forward scan current (NRE 1) for RC and Non-RC mode cited.

Scan Rate	Non-RC Mode (nA)	RC Mode (nA)	Amplification
10 mV/s	29.3	47.5	1.63
50 mV/s	43.7	59.3	1.36
100 mV/s	48.9	65.2	1.34
500 mV/s	64.9	86.6	1.30

**Table 2:** NRE 2 collection efficiency.

Scan Rate	*i*_2,a_ (nA)	*i*_1,c_ (nA)	*Φ*_2_
10 mV/s	42.3	46.5	90.7%
50 mV/s	40.6	61.8	65.7%
100 mV/s	42.7	70.5	60.6%
500 mV/s	47.1	110.5	40.2%

## Data Availability

Not applicable.
